# Microhardness and Chemical Composition of Different Metallic Brackets: An *In Vitro* Study

**DOI:** 10.3390/dj11090202

**Published:** 2023-08-24

**Authors:** Marine Colmant, Paul Fawaz, Kenneth Stanton, Oisin MacMichael, Bart Vande Vannet

**Affiliations:** 1 Faculty of Dentistry, Department of Orthodontics, University of Lorraine, 54000 Nancy, Francebart.vande-vannet@univ-lorraine.fr (B.V.V.); 2 Faculty of Mechanical and Materials Engineering, University College Dublin, D04 V1W8 Dublin, Ireland

**Keywords:** orthodontic brackets, hardness, chemistry, alloys, enamel

## Abstract

The purpose of the study is to compare the hardness of different metallic brackets to enamel and to determine their chemical composition and microstructure. Five metallic brackets (0.022″ × 0.028″ inch) from seven orthodontic firms produced in different alloys (Discovery^®^ Smart/Empower^®^/Genius^®^2 Metal/Victory^TM^ series/Equilibrium^®^/Damon Q) were chosen (*n* = 35). The hardness of the brackets and enamel was measured using a microhardness tester. The study of the chemical composition of brackets was carried out on a single bracket per series. A scanning electron microscope (SEM) equipped with an energy dispersive spectroscopy (EDS) detector was used. Analysis of the chemical composition of metallic brackets was obtained with Oxford Instruments Ultim Max Aztec software. Metallic brackets’ hardness varied from 203 to 439 HV. A significant difference exists between Ti Equilibrium^®^ brackets, the rest of the brackets and the enamel (*p*-value = 0.003). No significant difference was found between SS_a_(stainless-steel alloy), Co-Cr_a_(Cobalt-Chrome) brackets and the enamel. The chemical study confirms that the alloys used to produce metallic brackets validate all the data of the manufacturers except for Genius^®^2 Metal; according to this study, they are considered to be Co-Cr_a_ alloys. The study of the composition of brackets made it possible to confirm manufacturers’ data. Genius^®^2 Metal brackets, Empower^®^2 and Victory^TM^ series brackets filled the properties needed for orthodontic treatment. The hardness of metallic brackets is influenced by the alloy used and manufacturing method. Co-Cr_a_ brackets with hardness comparable to enamel can be considered as an alternative to SS_a_ brackets in patients allergic to nickel.

## 1. Introduction

Metals used in the manufacturing of orthodontic brackets are mostly alloys. Four major alloys are widely used: stainless steel alloy (SS_a_), chrome-cobalt alloy (Co-Cr_a_), titanium alloy (Ti_a_) and precious metal (PM_a_) [1].

An ideal bracket must fulfill the following criteria: biocompatibility, stability, mechanical properties meeting our treatment demand, non-iatrogenic fitting and removal, aesthetic and non-abrasive to enamel [2]. However, no material currently used can guarantee all these characteristics at once. Most orthodontic brackets are made from SS_a_. The latter are alloys of iron and carbon (Fe-C), containing, according to the ISO NF EN 10088-1 standard, at least 10.5% chromium and a carbon content of less than 2% [3]. However, the presence of nickel in SS_a_ and its release in vivo [4] can cause reactions ranging from allergy to cytotoxicity [5]. In Europe, this sensitivity to nickel is more frequent in women (10 to 15%) than in men (1 to 3%) [6]. The allergic response to nickel is independent of the amount of metal, but it seems to be caused more by skin contact than intraoral contact [7]. The alloy used and the implementation techniques influence the hardness of brackets.

Co-Cr_a_, also biocompatible and free of nickel, can replace SS_a_ brackets in patients allergic to this component [8]. The former has the advantage of producing little galvanic corrosion and presenting a polished surface. However, Co-Cr_a_ have friction properties less favorable compared to Ti or SS_a_ brackets.

Ti brackets have the advantage of presenting mechanical properties equivalent to SS_a_, with better corrosion resistance, excellent biocompatibility, and the absence of nickel-release [8]. Also, these brackets have a rough surface, which increases the risk of plaque formation [2].

Metal brackets can be coated with PM such as gold, platinum, or palladium. They are generally used in lingual techniques or for patients allergic to nickels [2].

The Vickers hardness (HV) of enamel has been studied in many publications. Warkentin et al. found microhardness values ranging from 270 to 420 HV for a maxillary molar [9]. These results are close to those found by Guttiérrez-Salazar and Reyes-Gasga varying between 270 and 360 HV, the lowest values being associated with the cervical area [10]. Similarly, Sa et al. [11] found hardness values for healthy enamel of 316 HV, close to that obtained by Aydin et al. of 330 HV [12], whereas it was only 39 HV for hypomature enamel with amelogenesis imperfecta. Enamel hardness is also reduced when the enamel is hypomineralized (HME) [13]. The HV of the brackets varies according to the studies, the alloys used, and the manufacturing method. Ideally, the enamel should be harder than the brackets to avoid wear of the teeth in contact [14]. Nevertheless, the wings of the bracket have to avoid plastic deformations and thus allow the bracket to support and transmit the forces coming from the orthodontic archwire [15]. The hardness of the bracket’s base should also be lower than that of the wings and the enamel in order to facilitate the debonding of the brackets at the end of treatment [16].

To our knowledge, no study has yet evaluated the microhardness and chemical composition of different metallic brackets. The null hypotheses are (a) no difference between the microhardness of different metallic brackets, and (b) no difference between the evaluated chemical composition and the one described by the manufacturer for different metallic brackets.

## 2. Materials and Methods

Seven different metallic brackets 0.022″ × 0.028″ inch produced in different alloys were chosen (Table 1) for a total sample of n = 35. Brackets for the left maxillary central incisor were chosen. Enamel and bracket hardness were measured with the same technique. These measurements were recorded on the buccal side near the edge of a lateral incisor and on the cuspid tip along the long axis of a canine for the enamel; those teeth were freshly extracted (less than 2 months) and conserved in 0.1%NaOCL (pH 10) [12]. Ethical approval for this study was granted (BUN 143.201.732.912).

In vitro manipulations and observations were performed at University College Dublin, School of Mechanical and Materials Engineering by a single-blinded engineer. A randomly chosen number was associated with each type of bracket.

The HV measurements of the brackets and enamel were carried out using a Buehler MicroMet 5101 microhardness tester (BUEHLER, Lake Bluff, IL, USA). Handling is carried out according to standard NF EN ISO 6507-1 [17].

**Figure 1:**
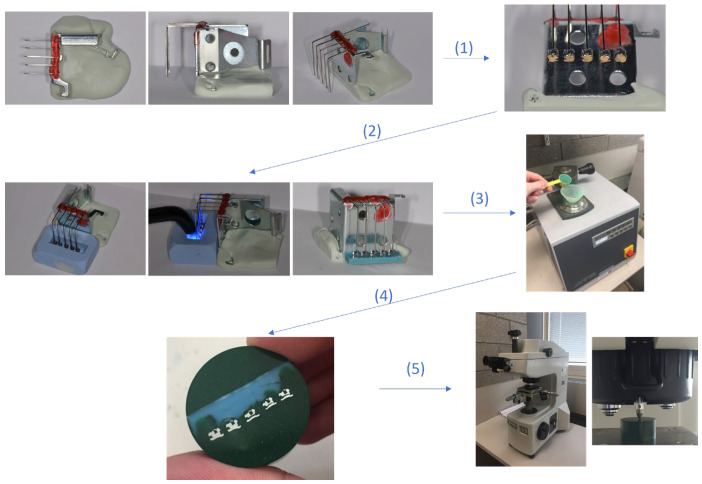
Summary of the randomized technique used to position the studied brackets. 1. Mounting of the different studied brackets 2. Polymerisation procedure. 3. Molding in flat silicone base. 4. embedding in bakelite. 5. testing of micro-hardness and microstructure.

The Vickers indentations were all performed with a 300 g load and a contact time of 10 s. Brackets were inserted into a positioning device equipped with stainless steel archwire 0.019″ × 0.025″ inch so as to be positioned at the same angulation, perpendicular to the arch wire, and presenting the same distal side to be tested. Then, they were molded into a flat silicone base using Optiband Ultra© light-curing compomer resin (Ormco, Glendora, CA, USA) (Batch no. 7.515.473) to create a flat resin strip, grouping together brackets from the same series. Each ruler is marked with a number to distinguish it from the other types of brackets tested, and within the same ruler, the brackets are numbered from 1 to 5. Subsequently, to perfect the support of each sample in the hardness tester, to ensure the flatness of each bracket and that all the samples were tested at the same level, the brackets were embedded in bakelite embedding resin (Multifast verte, Struers, Denmark). To achieve this, bakelite pellets were put into an automatic hot mounting press (MetPrep-OPAL 410, Coventry, UK). Finally, after cleaning and polishing we obtained the final base support of the tested brackets (Figure 1). This same fixation technique was also used to study the hardness of the enamel. Each sample grouping all the brackets of a bracket type is fixed on the test stage of the micro-hardness tester, and the surface is focused using the tester’s first objective lens (10×) to position the indenter next to the bracket tested. Then, each bracket is indented by the diamond-tipped indenter 5 times. The location of each indentation on each bracket was repeated exactly the same. Finally, the indentation was read via the higher-focusing objective lens (50×) to measure the diagonals of the indentation. These measurements were calculated to determine the hardness of the tested bracket.

The chemical compositions provided by the manufacturers were collected.

Subsequently, the microstructure was studied on all the brackets that underwent microhardness tests. The study of the chemical composition of brackets was carried out on a single bracket per series. These measurements were made on their distal face, which is most often flat and wide compared to the generally convex occlusal face.

Chemical composition analysis of orthodontic brackets was carried out using a scanning electron microscope (SEM) equipped with an energy dispersive spectroscopy (EDS) detector. The examination of a sample with the SEM does not require any specific preparation, but does however require a conductor of electricity sample, otherwise it gradually accumulates electrical charges during observation [18]. The microscope used is the Hitachi Regulus 8230 coupled with the integrated Regulus study software. The acceleration voltage is 30 kV.

Each specimen was tested twice at two separate randomly selected locations in the center of the bracket on the anterior side. The software used for the analysis of the chemical composition of metal orthodontic brackets is Oxford Instruments Ultim Max Aztec software, (v 5.0 Sept 2020).

The microstructure analysis was carried out using an optical microscope (Nikon Optiphot, Melville, NY, USA). These manipulations were carried out according to standard NF A05-150. For SS_a_, the surface treatment consists of an electrochemical attack with oxalic acid (Oxalic Acid Product Code 15.684.150, Honeywell-Fluka, Morris Plains, New Jersey) at 10% under 5 V for 20 s. Co-Cr_a_ are also treated by electrochemical attack with 3% hydrochloric acid (Hydrochloric Acid 1.18 SA, Romil Ltd., Cambridge, UK) at 3 V for 10 s. The surface treatment of Ti consists of a chemical attack with Kroll’s reagent (Kroll’s Reagent (192), Waterfall and O’Brien Ltd., Bristol, UK) (92% water, 6% nitric acid and 2% hydrofluoric acid) for 20 s [19].

### Statistical Tests

Statistical analyses were performed using IBM Statistics SPSS 26 software (Armonk, NY, USA). The level of significance was set at *p*-value ≤ 0.05 for all statistical analyses. The primary outcome variable of the study was Vickers hardness (HV). The appropriate alpha error was set at 0.05 and the beta error was set a 0.20. Using the data form a previous pilot study, a sample size of 30 would detect a significant difference in HV between different metallic brackets. To compare HV of metal orthodontic brackets, two normality tests, Kolmogorov–Smirnov and Shapiro–Wilk, were performed. They show that the HV values do not follow a normal pattern. Therefore, a non-parametric Kruskal–Wallis test was performed to compare the HV:

Between the different types of alloys used for the manufacturing of orthodontic brackets tested: SS_a_, Co-Cr_a_, Ti, and with enamel.

Between the different SS brackets: Damon Q, Empower 2, Victory series and Discovery smart.

A non-parametric Mann–Whitney test was performed to compare the HV of Co-Cr_a_, Topic^®^ (Dentaurum, Ispringen, Germany), Genius^®^2 metal brackets (Dental Technology, Tainan city, Taiwan) and conventional and self-ligating brackets. Bonferroni’s post hoc test is used to determine significant differences between each group. Intra-observer reproducibility was assessed using the intraclass correlation coefficient (ICC) with a 95% confidence interval; the ICC for all measurements was greater than 0.900, which indicates excellent reproducibility.

## 3. Results

**Figure 2:**
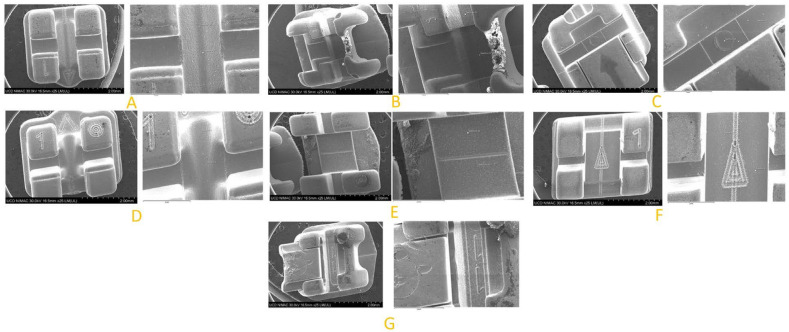
Different bracket types viewed on SEM and their chemical composition study point. (**A**) Discovery^®^ Smart (Dentaurum, Ispringen, Germany). (**B**) Empower^®^2 (American Orthodontics, Sheboygan, WI, USA) (**C**) Genius^®^2 Metal (Dental Technology, Tainan city, Taiwan). (**D**) Topic^®^ (Dentaurum, Ispringen, Germany) (**E**) VictoryTM series (3M UniteK™, Monrovia, CA, USA). (**F**) Equilibrium^®^ (Dentaurum, Ispringen, Germany). (**G**) Damon Q (Ormco, Glendora, CA, USA).

Seven different brackets brands were tested (Table 1). For each type of bracket, five brackets were tested, with five measurements per bracket. There was therefore a total of 25 measurements per type of bracket. Results obtained are summarized in Table 2. The different bracket types were viewed on SEM and their chemical composition studied (Figure 2).

In the SS_a_ group, there is a significant difference in microhardness between the [A] brackets produced with the metal-injection-molding (MIM) technique and the [E] brackets produced by milling (*p*-value = 0.014).

Statistical analysis showed a significant difference between the microhardness of enamel and [G] brackets (*p*-value = 0.003). Also, there is a significant difference in microhardness between all the SS_a_ brackets (*p* value < 0.05), except between the body of the [B] brackets and the [E] brackets (*p* value > 0.05).

The hardness of the [E] brackets (389.6 ± 3.2 HV) is greater than that of enamel (331 ± 60 HV) without being significantly different (*p*-value = 0.090).

The hardness of [A] brackets (326 ± 9 HV) is similar to enamel (331 ± 60 HV). Statistical analysis shows the absence of significant differences between these attachments and enamel (*p*-value = 0.0549).

Bracket [B] has two very different microstructures within the tested distal part, one of which is harder than the other. The so-called “hard” part corresponds to the main body while the so-called “soft” part corresponds to the base of the bracket. Their microhardness values, respectively 387.7 ± 5.7 HV and 275.7 ± 13.8 HV, are significantly different (*p* value = 0.009). Nevertheless, the values found at the level of the body of the bracket are similar to (and slightly higher than) the value of the enamel without a statistically significant difference (*p* value > 0.05).

The hardness of [G] brackets is 439 ± 11 HV. These are the highest hardness values among the brackets tested. Statistical analysis shows a significant difference between their microhardness and that of the enamel and other SS_a_ orthodontic brackets (*p* value < 0.05).

**Figure 3:**
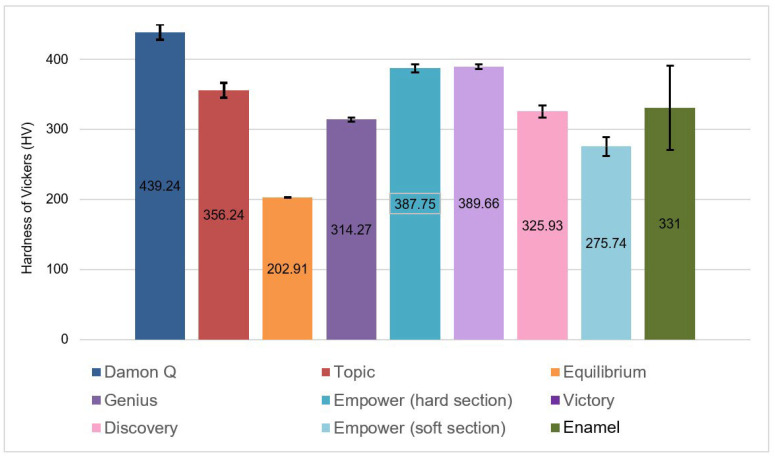
Vickers hardness of different metallic brackets compared to the enamel.

The microhardness of [D] brackets is 356 ± 11 HV, and that of the [C] brackets is 314 ± 3 HV. Both are lower than the enamel values (393 ± 21 HV). Statistical analysis confirms no significant difference between the microhardness of Co-Cr_a_ brackets and the enamel (*p*-value = 0.568). Metallic brackets have a hardness varying from 203 to 439 HV against 331 ± 60 for enamel. Statistical analysis showed a significant difference between Ti [F] brackets with the rest of the brackets and the enamel (*p*-value = 0.003). Also, the analysis did not show any significant difference between SS_a_, Co-Cr_a_ brackets and the enamel (Figure 3).

However, there is a significant difference in the microhardness of the Co-Cr_a_ brackets with the Ti brackets (*p* value = 0.005).

Concerning the comparison of Co-Cr_a_ brackets, between them, [D] and [C], Mann–Whitney’s analysis shows a significant difference (*p*-value = 0.009).

The hardness of [F] brackets is 203 ± 1 HV and is significantly softer than enamel and other orthodontic brackets (*p* value = 0.005).

The study of the composition confirms that the alloy used to produce the [E] is a SS_a_. The alloy used to produce [A] is a stainless-steel alloy rich in iron, carbon, chromium and nickel. [B] brackets are also produced with SS_a_ as stated by the manufacturer.

**Figure 4:**
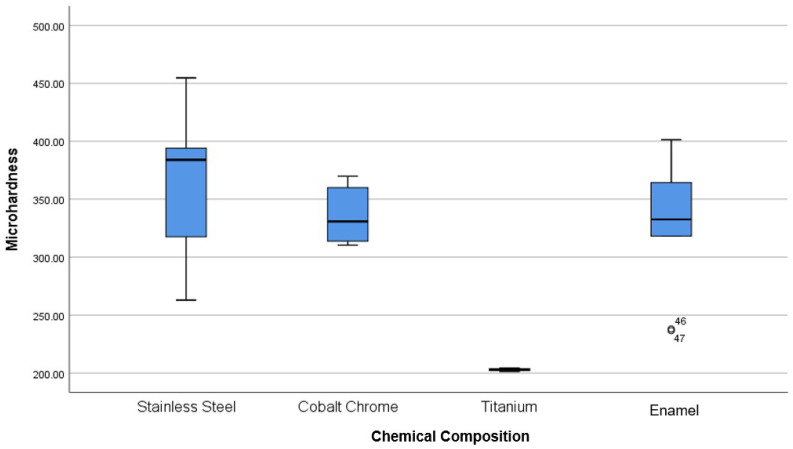
Comparison of the microhardness of different metallic brackets according to their chemical composition with each other and with the enamel. 4.6 first lower molar. 4.7 s lower molar.

Brackets [G] are made of SS_a_. It was not possible to compare the data collected during the study with the manufacturer’s data (Figure 4).

Bracket [C] is composed of the Co-Cr_a_. Its composition contains molybdenum and aluminum, and traces of silicon, manganese, titanium and iron, apart from the carbon traces found. This was not in accordance with the manufacture’s data, which stated that it was a SS_a._

Bracket [D] is composed of Co-Cr_a_, which is consistent with the data provided by the manufacturer (Table 3).

Bracket [F] is produced in pure Ti despite the presence of carbon and oxygen contaminants and traces of aluminum and iron (Table 3).

**Figure 5:**
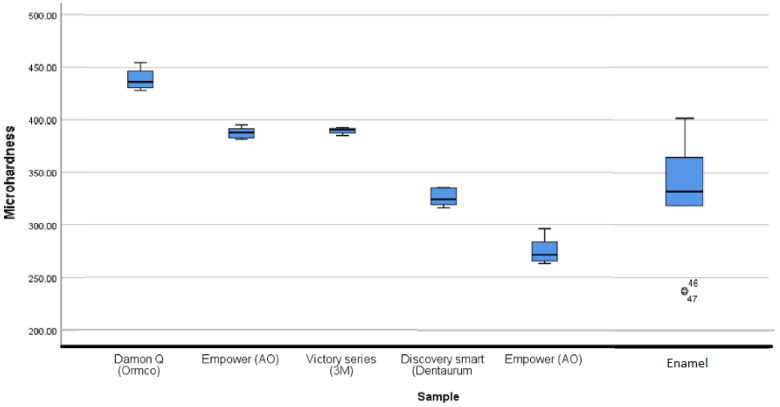
Comparison of the microhardness of different stainless-steel brackets to each other and to the enamel. 4.6 first lower molar. 4.7 s lower molar.

**Figure 6:**
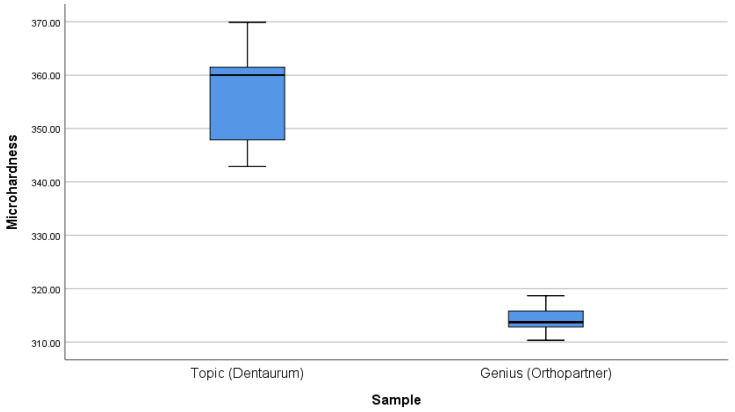
Comparison of the microhardness of different chrome-cobalt brackets to each other.

Microhardness results are summarized in and Figure 2, Figure 3, Figure 4, Figure 5 and Figure 6.

Chemical composition results are summarized in Table 3.

## 4. Discussion

The hardness of metals is determined by their chemical composition, internal structure, and manufacturing technique. The study of the chemical composition and microstructure of various metallic brackets makes it possible to apprehend the values of hardness as well as to confirm the data announced by the manufacturers and published in the literature. The study of the composition of orthodontic brackets [G], [B], [E], and [A] confirms the data from manufacturers announcing the use of a SS_a_ and is in agreement with the data from the literature [20,21].

**Figure 7:**
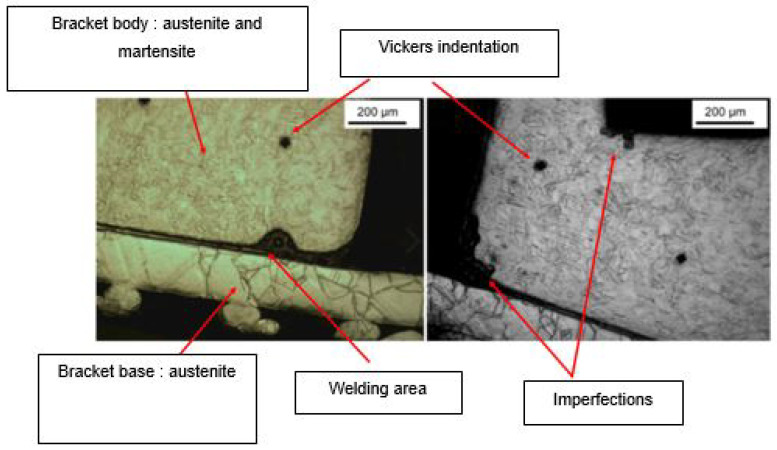
Microstructure of Victory^TM^ series brackets.

**Figure 8:**
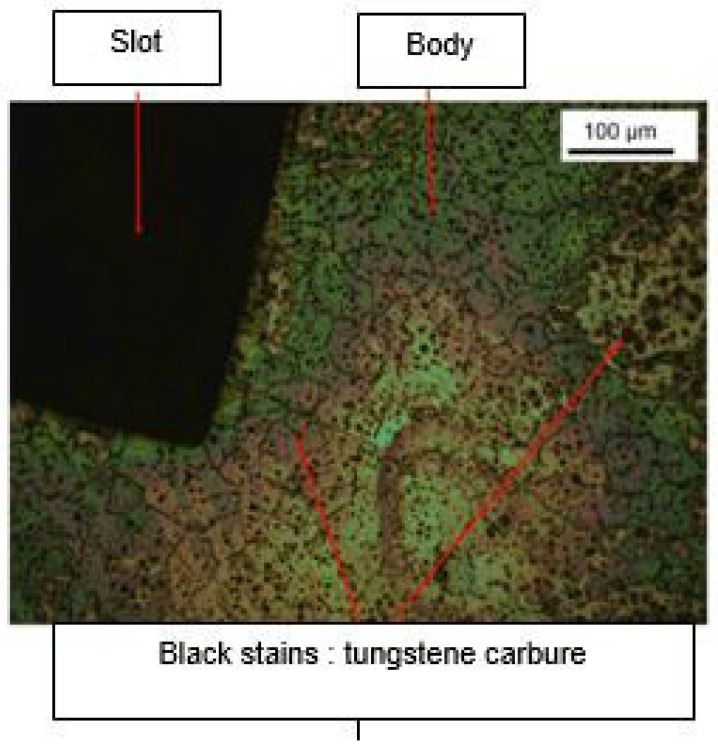
Microstructure of Discovery^®^ Smart brackets.

**Figure 9:**
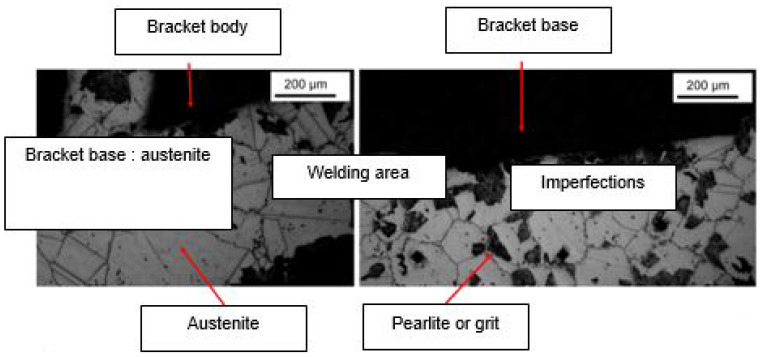
Microstructure of Empower^®^ 2 brackets.

**Figure 10:**
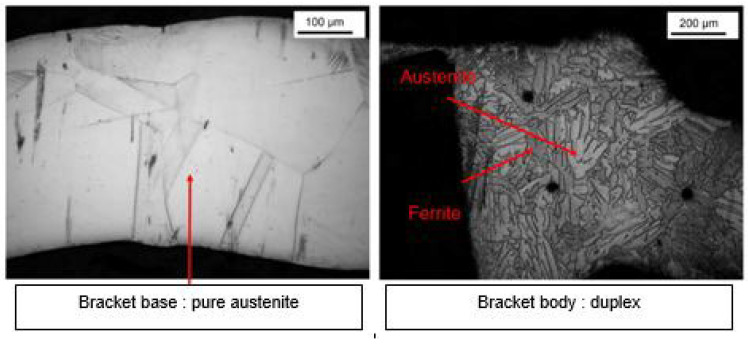
Microstructure of Damon^®^ Q brackets.

**Figure 11:**
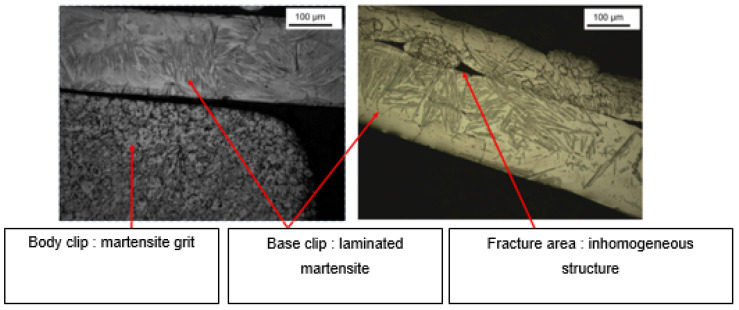
Microstructure of a Topic^®^ bracket.

**Figure 12:**
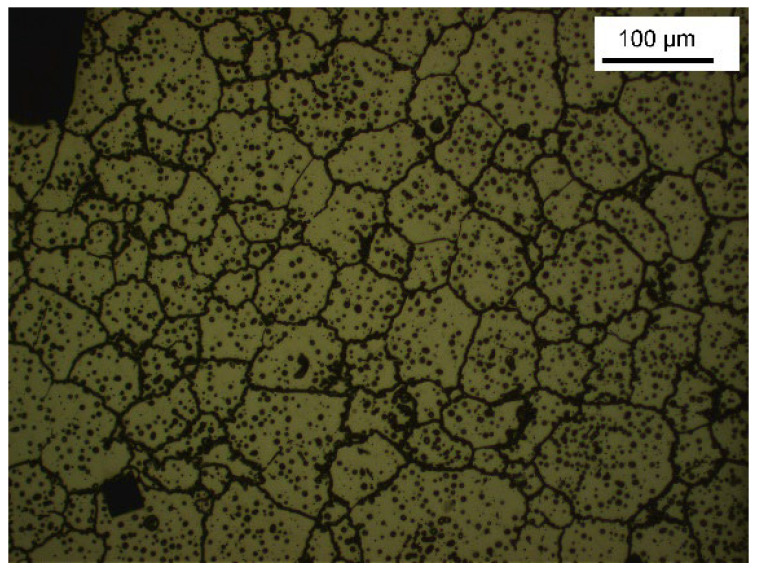
Microstructure of a Genius^®^2 Metal bracket.

**Figure 13:**
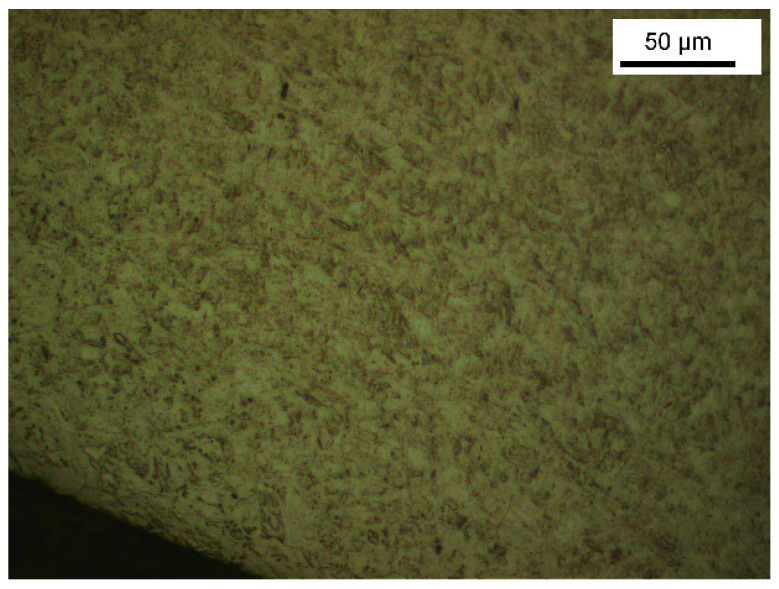
Microstructure of an Equilibrium^®^ bracket.

**Figure 14:**
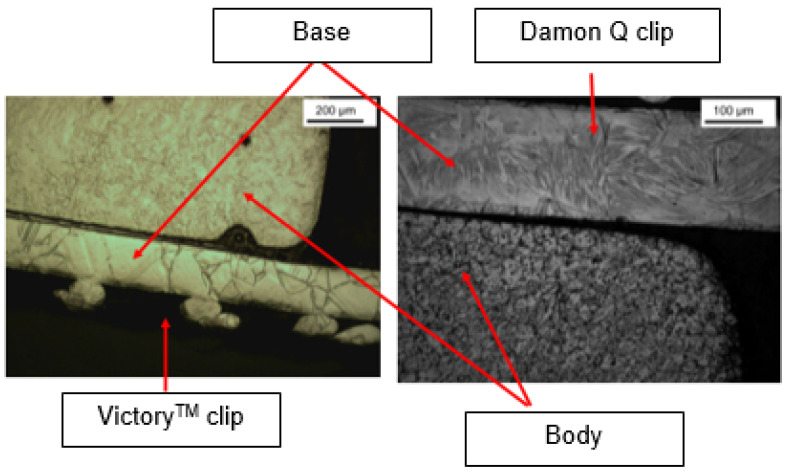
Shows the differences in microstructures within Damon^®^ Q and Victory^TM^ brackets.

Microstructure analysis revealed structural differences within the SS_a_ brackets. Brackets [A] have the same microstructure, which suggests that they are produced using a one-step casting method such as the MIM method stated by the manufacturer, resulting in a monolithic part. Unlike the latter, [G], [B] and [E] brackets have differences within their structure, between their body and their base. Figure 7 shows the microstructure of Victory^TM^ brackets in SSa with differences between body and base. Figure 8 shows the microstructure of Discovery^®^ brackets also in SSa. Figure 9 with Microstructure of Empower^®^ 2 brackets in SSa (austenite). Figure 10 Microstructure of Damon^®^ Q brackets in SSa and pure austenite is shown. Figure 11 shows the microstructure of a Topic^®^ bracket (CoCr). Figure 12 depicts the microstructure of a Genius^®^2 Metal bracket (SSa). Figure 13 shows the microstructure of an Equilibrium^®^ bracket in Titanium and Figure 14 shows the differences in microstructures within Damon^®^ Q and Victory^TM^ brackets. (Figure 7, Figure 8, Figure 9, Figure 10, Figure 11, Figure 12, Figure 13 and Figure 14).

This difference in microstructure may be due to:

- A different thermal treatment depending on the structures of these brackets; however, the small size of the sample makes this hypothesis unlikely.
- The assembly by the welding of two structures from different production paths, which disagrees with the manufacturers’ data. According to the latter, the [E] brackets are produced by a milling method and the [B] brackets are produced by the MIM (metal-injection-molding) method, normally allowing a monolithic bracket to be obtained.

Brackets [B] have been subject to a micro-hardness study of their base in addition to their body. It presents a body whose microstructure confers superior mechanical properties compared to those of the base, since it presents a significantly higher hardness than that of the base, while the hardness of the latter is significantly lower than enamel. This agrees with the data from the literature because it facilitates their removal at the end of the treatment. In the case of [B] brackets, there is no significant difference in their microhardness compared to that of enamel. Also, the “similar” microstructures of [G] and [E] brackets may suggest a similar configuration.

Hardness values for [E] brackets are 389.7 ± 3.3 HV. These results are in agreement with those found in the literature [21].

Brackets [G] have a particular microstructure such as martensitic SS_a_ that have the characteristics of presenting high mechanical resistance [22,23], which is reflected in the high microhardness values being the highest among all brackets tested.

The hardness associated with [A] brackets (326 ± 9 HV) is greater than that usually associated with austenitic SS_a_, which tend to exhibit relatively poor mechanical properties [22,23]. This difference may be caused by a defect in the micro indentation test related to the heterogeneity of the microstructure of these brackets as stated previously.

Brackets [A] (type 316 stainless steel), [B] (duplex SS) and [E] (type PH17-7 or 631 SS) have increasing carbon contents associated with increasing microhardness values. Indeed carbon is mainly used to reinforce materials, so a lower content generally means lower mechanical properties [17]. Bracket [G] (Type 420 stainless steel) deviates from this rule as they have the lowest carbon content but the highest microhardness values among the SS_a_ brackets tested. These observations show that the microhardness and more generally the mechanical properties of metal orthodontic brackets are conditioned by their composition but also by their structural arrangement. Also, these data are in disagreement with Matasa et al. for whom, the more the number of the AISI classification increases, the more the carbon content tends to decrease and the hardness tends to increase [24].

The hardness of the tested SS_a_ brackets averages at 363 ± 60 HV. The latter is no different from that of Co-Cr_a_ brackets nor enamel but is significantly different from that of Ti brackets (*p*-value < 0.05), which is not always the case in the literature [25].

Regarding the influence of the bracket production method, it appears that within the group of SS_a_ brackets, [A] brackets produced using a MIM method have a lower hardness than [E] brackets produced by milling. These results are in agreement with data from the literature [3].

Nevertheless, the absence of data concerning [G] brackets and the microscopic observation of [B] brackets not confirming their production in the MIM technique requires additional studies to extend this hypothesis to the advantages of orthodontic brackets.

The study of the composition (presence of tungsten) and the microstructure (small, condensed grains) suggest a high mechanical resistance of [D] brackets, which is reflected in HV = 356 ± 10.6. Indeed tungsten is known for its very high hardness [26]. Zinelis et al. also studied them and observed a similar composition but a lower HV [20].

The study of the composition of [C] brackets shows that they are produced in Co-Cr_a_, which disagrees with the manufacturer’s data describing it as an SS_a_. In addition, unlike [D] brackets, they are rich in carbon, which may explain their lower hardness (314 ± 3 HV). The HV values of the Co-Cr_a_ brackets found in the present study are in agreement with the data of the Francophone Society of Dental Biomaterials [21]. Despite a significantly different hardness between the two Co-Cr_a_ brackets tested, the latter generally have a hardness comparable to enamel (335 ± 23 HV versus 331 ± 60 HV for enamel), which makes them less traumatic for the enamel of the opposing teeth.

The study of [F] brackets confirms that they are produced in pure titanium: it has the lowest hardness among all the brackets tested (203 ± 1 HV) and the values found in the present study seem to be close to those found in the literature [21,27]. Ti brackets have a hardness significantly lower than the hardness of enamel. This allows them to be non-iatrogenic with respect to the enamel, which makes them particularly interesting in patients with impaired enamel (amelogenesis imperfecta, HME), severe overbite, or suffering from bruxism. However, this hardness seems to be insufficient to withstand the stresses of orthodontic archwires in NiTi (300 to 430 HV) and SS_a_ (600 HV). This can be the cause of wear and plastic deformation of the groove of the brackets preventing full engagement of the archwire and optimal dental movement [27].

The study of the composition of brackets also made it possible to confirm manufacturers’ data. Brackets [C] have good mechanical properties in addition to being nickel-free and having a high chromium content, a sign of good corrosion resistance. Brackets [B] have good mechanical properties, low nickel content, and high chromium content. However, their high hardness (387.7 ± 5.7 HV) can be harmful to the antagonist enamel, and to the enamel of the canine tips (237 ± 1 HV). Brackets [E] are comparable to brackets [B] but their lower chromium content suggests that their corrosion resistance is slightly reduced compared to the latter. Brackets [G] have a low nickel content but have the disadvantage of being harmful to the enamel given their high hardness and low chromium content. The absence of nickel in [D] brackets is beneficial from a biological point of view, but their microstructures make their breaking strength very poor. Finally, brackets [F] are also nickel-free and their pure titanium composition suggests they have very satisfactory corrosion resistance. Their low hardness may be insufficient to withstand the constraints of orthodontic archwires, but it interesting for patients where enamel integrity is essential. Overall, the different types of metal alloys used by the manufacturers in our sample are known and approved medically to be biocompatible: according to the present study most have a high hardness and therefore a high mechanical resistance.

No specific preparation of the sample is carried out because only the elementary chemical composition of the bracket was sought to determine the alloy used. It was therefore unnecessary to carry out a polishing generally carried out when a high-resolution analysis is desired.

In this study, Vickers indentations were all performed with a load of 300 g and a contact time of 10 s. These experimental conditions are similar to the conditions used in past studies on the hardness of orthodontic brackets [20,28].

**Figure 15:**
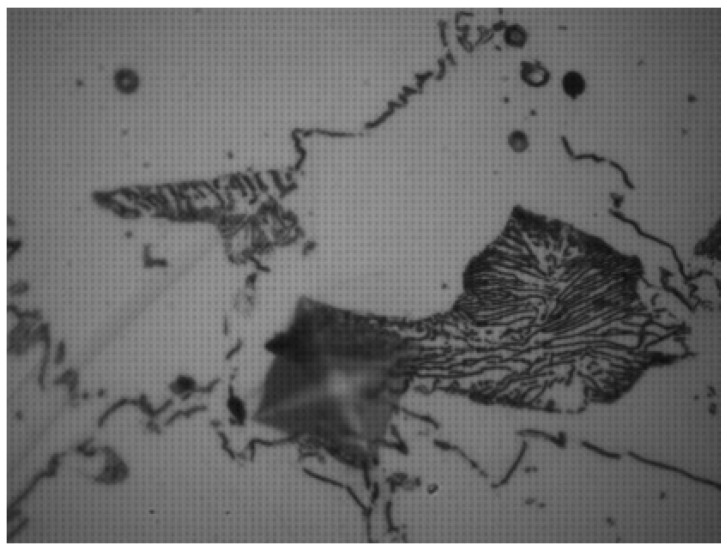
Example of indentation on a Discovery^®^ Smart clip half on the bottom structure and on a pearlite or grain.

Nevertheless, it is important to note the limitations of our study; the use of a non-automatic microhardness tester (like the one used in our study) can cause errors as each surface of the sample and each measurement are dependent on the experience of the observer (with his naked eye). This can introduce focusing and measurement errors. The use of an automatic microhardness tester would limit this type of error. Also, the alloys used for the manufacturing of the sample are not homogeneous. The indentation can thus take place partially or totally on grains or pearlites at the origin of heterogeneous results (Figure 15). Ideally, an indentation should be made on a completely homogeneous region.

Metallography is highly dependent on the experience of the observer. In the present study, an experienced engineer performed the sample preparation. This preparation allowed him to observe very precisely the microstructure of metals. It is important to mention that the sample number should be taken into consideration when analyzing the results obtained. Hence, further studies with larger sample size might be important to confirm our results.

## 5. Conclusions

Our null hypotheses have been rejected; the hardness of metal orthodontic brackets is influenced by the alloy used and the manufacturing method.

Nickel-free Co-Cr_a_ brackets with hardness comparable to enamel can be considered as a serious alternative to SS_a_ brackets in patients allergic to nickel.

All brackets tested in this study are considered harmless to the enamel with SS_a_ brackets having the highest HV = 362.58 ± 59.58, then Co-Cr_a_ brackets with a HV = 335.35 ± 23.47 and the lowest was Ti brackets with a HV = 202.91 ± 1.26.

## Figures and Tables

**Table 1:** Summary of the manufacturing methods of the orthodontic brackets tested (manufacturers’ data).

*Brackets*	*Manufacturing*	*Alloy*	*N*
*Discovery^®^ Smart (Dentaurum, Ispringen, Germany) = A*		*SS_a_*	*5*
*Empower^®^2 (American Orthodontics, Sheboygan, WI, USA) = B*	*Metal Injection Molding*	*SS_a_*	*5*
*Genius^®^2 Metal (Dental Technology, Tainan city, Taiwan) = C*		*SS_a_*	*5*
*Topic^®^ (Dentaurum, Ispringen, Germany) = D*		*Co-Cr_a_*	*5*
*Victory^TM^ series (3M UniteK™, Monrovia, CA, USA) = E*	*Milling method*	*SS_a_*	*5*
*Equilibrium^®^ (Dentaurum, Ispringen, Germany) = F*		*Ti*	*5*
*Damon Q (Ormco, Glendora, CA, USA) = G*	*No information provided by the manufacturer*	*SS_a_*	*5*

**Table 2:** Microhardness (HV) of enamel and orthodontic brackets tested.

Microhardness (HV)	Mean	95% Confidence Interval Mean for (Lower/Upper) Bound	Standard Deviation	Minimum/Maximum
SS_a_	362.58	337.42/387.74	59.58	262.91/454.65
Co-Cr_a_	335.35	318.56/352.14	23.47	310.32/369.92
Ti	202.91	200.89/204.92	1.26	201.22/204.28
Enamel	330.52	284.58/376.45	59.75	236.6/401.3

**Table 3:** Summary table of chemical composition results.

	Percentage (%)																				Alloy	Vickers (HV)	Microstructure
		C	O	Na	Al	Si	P	Cr	Mn	Fe	Ni	Cu	Ti	F	S	Ca	Mo	Co	Sr	W			
**VICTORY**	Average	43	20	<1	4	0.5		10		20	2	0.4									Stainless steelPH 17-7 (AISI)	390	Base and body same SS but different thermal treatment.Imperfections at the base–body junction due to the welding material.
	σ	5	<1	0.2	0.4	0.2		0.5		2	0.5												
**DISCOVERY**	Average	16			0.3	0.3		15		57	9	2									Stainless steel316 (AISI)	326	Heterogeneous but identical microstructure between base and body.
	σ	6				0.06		<1		5	0.5	0.2											
**EMPOWER**	Average	27	7		0.8	0.5		14		45	2.5	3				0.1					Stainless steelDuplex	380 (corps) 276 (base)	Body: duplex SS (austenite and ferrite). Base: austenite SS.
	σ	15	6		0.8			<1		20	1	0.5											
**DAMON**	Average	14	23		0.1	7		10		40	2	1.3		2.5	0.04	0.1					Stainless steel420 (AISI)	439	Base and body: different thermal treatments. Base has flaws.
	σ	1.5	3			<1		1		4.5	0.1	0.1		0.6									
**GENIUS**	Average	55			2.2	1		16	0.4	0.2			0.6				4	20			Co-Cr	314	Black spots: carbon
	σ	3.6			0.1	0.06		<1	0.1								0.4	2					
**TOPIC**	Average	17			0.3	1.7	0.3	21.5										43	3	13.5		356	Small grains: sintered material under high tension/coloring: acid surface treatment
	σ	4				0.3	0.2	5.3										12	1.4	12			
**EQUILIBRIUM**	Average	5.5	10		0.1					0.06			85								Pure titanium	203	Homogeneous structure
	σ	0.4	1.6										2										

## Data Availability

All data are available upon request.

## References

[1] Brantley W., Berzins D., Iijima M., Tufekçi E., Cai Z. (2017). Structure/property relationships in orthodontic alloys. Orthodontic Applications of Biomaterials.

[2] Khan H., Price S. (2015). Orthodontic Brackets: Selection, Placement and Debonding.

[3] (2014). Aciers Inoxydables—Partie 1: Liste des Aciers Inoxydables.

[4] Eliades T., Zinelis S., Eliades G., Athanasiou A.E. (2002). Nickel content of as-received, retrieved, and recycled stainless steel brackets. Am. J. Orthod. Dentofac. Orthop..

[5] Shintcovsk R.L., Knop L.A.H., Gandini L.G., Martins L.P., Pires A.S. (2015). Comparison surface characteristics and chemical composition of conventional metallic and nickel-free brackets. Braz. Oral Res..

[6] Beaufils S., Daltin A.-L., Millet P. Alliages non précieux (hors titane et ses alliages). https://www.em-consulte.com/article/1072780/resume/alliages-non-precieux-hors-titane-et-ses-alliages.

[7] Szustakiewicz B., Djerbi N., Gondy A., Derki M., Devulder-Salana A., Pindi G., Rakotomalala H., Sergent O., Swead M., Tronet J. (2009). Behavior of materials in the oral environment. Orthod. Fr..

[8] Malinowski S., Moutaabbib L. (2009). Metal brackets. Orthod Fr..

[9] Warkentin M., Freyse C., Specht O., Behrend D., Maletz R., Janda R., Ottl P. (2018). Correlation of ultrasound microscopy and Vickers hardness measurements of human dentin and enamel—A pilot study. Dent Mater..

[10] Del Pilar Gutiérrez-Salazar M., Reyes-Gasga J. (2003). Microhardness and chemical composition of human tooth. Mater. Res..

[11] Sa Y., Liang S., Ma X., Lu S., Wang Z., Jiang T., Wang Y. (2014). Compositional, structural and mechanical comparisons of normal enamel and hypomaturation enamel. Acta Biomater..

[12] Aydın B., Pamir T., Baltaci A., Orman M.N., Turk T. (2015). Effect of storage solutions on microhardness of crown enamel and dentin. Eur. J. Dent..

[13] Elhennawy K., Manton D.J., Crombie F., Zaslansky P., Radlanski R.J., Jost-Brinkmann P.G., Schwendicke F. (2017). Structural, mechanical and chemical evaluation of molar-incisor hypomineralization-affected enamel: A systematic review. Arch. Oral Biol..

[14] Patcas R., Eliades T. (2017). Enamel alterations due to orthodontic treatment. Orthodontic Applications of Biomaterials.

[15] Eliades T., Bradley T.G., Brantley W. (2017). Material properties and effects on mechanotherapy. Orthodontic Applications of Biomaterials.

[16] Iijima M., Zinelis S., Papageorgiou S.N., Brantley W., Eliades T. (2017). Orthodontic brackets. Orthodontic Applications of Biomaterials.

[17] (2018). Matériaux Métalliques—Essai de Dureté Vickers—Partie 1: Méthode d’Essai.

[18] Dupeux M. (2015). Aide Mémoire: Science des Matériaux.

[19] (1985). Produits en Acier—Techniques d’Examen Micrographique.

[20] Zinelis S., Annousaki O., Makou M., Eliades T. (2005). Metallurgical Characterization of Orthodontic Brackets Produced by Metal Injection Molding (MIM). Angle Orthod..

[21] Gregoire G., Grosgogeat B. (2010). Alliages Dentaires.

[22] Association ESSD (2007). Stainless Steel: Tables of Technical Properties.

[23] Murry G., Lévêque R. (2015). Aide Mémoire Métallurgie.

[24] Matasa C.G. (1992). Direct bonding metallic brackets: Where are they heading?. Am. J. Orthod. Dentofac. Orthop..

[25] Alavi S., Kachuie M. (2017). Assessment of the hardness of different orthodontic wires and brackets produced by metal injection molding and conventional methods. Dent. Res. J..

[26] Groover M.O. (2010). Chapitre 6: Metals. Fundamentals of Modern Manufacturing: Materials, Processes, and Systems.

[27] Eliades T. (2007). Orthodontic materials research and applications: Part 2. Current status and projected future developments in materials and biocompatibility. Am. J. Orthod. Dentofac. Orthop..

[28] Eliades T., Zinelis S., Eliades G., Athanasiou A.E. (2003). Characterization of as-received, retrieved, and recycled stainless steel brackets. J. Orofac. Orthop..

